# Extensive Injury to the Jaw, Following the Removal of an Incisor Tooth by the Key Instrument—Remarks on the Extraction of Deciduous Teeth

**Published:** 1851-07

**Authors:** Thomas Underwood

**Affiliations:** Honorary Dentist of the St. George's and St. James' Dispensary.


### ARTICLE XIII.

***Extensive Injury to the Jaw, following the Removal of an Incisor Tooth by the Key Instrument—Remarks on the Extraction of Deciduous Teeth.*** By Thomas Underwood, Esq. ***Honorary Dentist of the St. George's and St. James' Dispensary.***

The following case exemplifies the injury that may be done to the maxillæ by the two forcible extraction of the temporary teeth, particularly where there is a scrofulous tendency in the constitution.

The patient is a young lady, fourteen years of age, the appearance of whose face is very peculiar: the lower jaw is excessively pointed, presenting a sharp, wedge-like appearance, instead of the natural rounded form; and the following peculiarities are to be observed in the interior of the mouth: the four permanent incisors and left canine, in the lower jaw, are wanting, together with their alveoli; the right canine occupies the place of the right central incisor; and the bicuspids, or small molar teeth, branch off on each side. There are a small space of an eighth of an inch between the canine and left bicuspis, but the other teeth are in close contact. The jaw consequently contains nine, instead of fourteen, the usual complement of teeth at this age.

The account which I received from my patient and her friends was, that six years ago one of the temporary incisor teeth was removed by the key—an instrument, the use of which was evidently unnecessary for a temporary tooth, more especially a temporary incisor. After the extraction, an abscess formed under the chin, about half an inch from the symphysis, which ultimately pointed outwards, and continued discharging pus for four years, exfoliated bone being now and then thrown off. Granulations then sprang up, and the wound healed. The result of the injury was, that five of the front teeth, with their alveoli, and a portion of the lower maxilla, an inch and a quarter in length, have disappeared.

A tumor having lately again formed, in much about the same position as the former abscess, the medical man in attendance on the case, though he believed it to be an enlargement of the sub-maxillary gland, was nevertheless anxious that a dentist should examine the mouth. I was accordingly consulted, but found the teeth all sound, and my opinion coincided with his, that it was an enlargement of the gland, which, owing to the angular state of the jaw, appeared to be protruded into the cavity of the mouth. The tumor has almost disappeared under the use of the compound iodine ointment. The patient is evidently scrofulous, which circumstance will, in a great measure, account for the extensive injury which has occurred; yet the operator was much to blame who used such an instrument as the key, so rarely admissible, even in adult cases, to extract a temporary *incisor* tooth. The result has been a disfigurement, which no art can rectify ; but one or two lessons may be gathered from the case—viz. whenever we are called upon to remove deciduous teeth, the constitutional tendency of the little patient ought to be considered, and this is especially a matter of primary importance where any degree of force is to be used. Again, though parents, and even many practitioners, are in favor of extracting the temporary teeth, as soon as the permanent ones make their appearance, experience points to the contrary course; and I have invariably seen, in my practice, that the longer the first teeth can be retained in the jaw the better, for irregularity arising from their removal being deferred, can always be rectified; and contraction resulting from too early extraction is beyond our power. Even in cases where children suffer from tooth-ache of the temporary teeth, I have always found a simple fomentation of water, as hot as it can be borne, applied for half an hour to the outside of the cheek, opposite the seat of pain, together with a gentle aperient, sufficient to afford entire relief; and by this means the teeth may be retained until their successors displace them, or at least until they may be removed with very little force.—*Lancet.*

